# Supplementary material for: Can Whole-Body Baseline CT Radiomics Add Information to the Prediction of Best Response, Progression-Free Survival, and Overall Survival of Stage IV Melanoma Patients Receiving First-Line Targeted Therapy: A Retrospective Register Study
Source: Diagnostics (Basel). 2023 Oct 14;13(20):3210. doi: 10.3390/diagnostics13203210 (PMC10605712; doi:10.3390/diagnostics13203210)
Supplement: Supplementary file 1 [file diagnostics-13-03210-s001.zip › diagnostics-2605453-supplementary.pdf]

Supplement Table S1: CT scanner vendors and scan parameters.

Cohort	Scanner	Vendor	Collimation	Rotation time	Pitch	Slice thickness	Increment	Image reconstruction	Number of patients
**In-house**	SOMATOM Definition AS+	Siemens	64 × 0.6 mm	0.5 s	0.6	3	3	medium smooth kernel	6
	SOMATOM Definition Flash	Siemens	128 × 0.6 mm	0.5 s	1.0	3	3	medium smooth kernel	5
	SOMATOM Force	Siemens	128 × 0.6 mm	0.5 s	0.6	3	3	medium smooth kernel	39
	Sensation 64	Siemens	64 × 0.6 mm	0.5 s	0.6	3	3	medium smooth kernel	2
	Biograph128	Siemens	128 × 0.6 mm	0.5 s	0.8	3	3	medium smooth kernel	13
**External**	Aquillion One	Canon	n.a.	n.a.	n.a.	n.a.	n.a.	n.a.	4
	BrightSpeed	GE	n.a.	n.a.	n.a.	n.a.	n.a.	n.a.	3
	LightSpeed VCT	GE	n.a.	n.a.	n.a.	n.a.	n.a.	n.a.	3
	Emotion 16	Siemens	n.a.	n.a.	n.a.	n.a.	n.a.	n.a.	2
	Emotion 6	Siemens	n.a.	n.a.	n.a.	n.a.	n.a.	n.a.	1
	Sensation 16	Siemens	n.a.	n.a.	n.a.	n.a.	n.a.	n.a.	1
	SOMATOM Definition AS	Siemens	n.a.	n.a.	n.a.	n.a.	n.a.	n.a.	4
	SOMATOM Definition Edge	Siemens	n.a.	n.a.	n.a.	n.a.	n.a.	n.a.	2
	SOMATOM Definition Flash	Siemens	n.a.	n.a.	n.a.	n.a.	n.a.	n.a.	5
	SOMATOM Volume Zoom	Siemens	n.a.	n.a.	n.a.	n.a.	n.a.	n.a.	1
